# cDNA Cloning and Expression Analysis of Gustavus Gene in the Oriental River Prawn *Macrobrachium nipponense*

**DOI:** 10.1371/journal.pone.0017170

**Published:** 2011-02-16

**Authors:** Fengying Zhang, Liqiao Chen, Jianguang Qin, Weihong Zhao, Ping Wu, Na Yu, Lingbo Ma

**Affiliations:** 1 College of Life Science, East China Normal University, Shanghai, China; 2 Chinese Academy of Fishery Sciences, East China Sea Fisheries Research Institute, Shanghai, China; 3 School of Biological Sciences, Flinders University, Adelaide, Australia; University of Illinois at Chicago, United States of America

## Abstract

The gustavus gene is required for localizing pole plasm and specifying germ cells. Research on gustavus gene expression will advance our understanding of the biological function of gustavus in animals. A cDNA encoding gustavus protein was identified and termed *MnGus* in the oriental river prawn *Macrobrachium nipponense*. Bioinformatic analyses showed that this gene encoded a protein of 262 amino acids and the protein belongs to the Spsb1 family. Real-time quantitative PCR analyses revealed that the expression level of *MnGus* in prawn embryos was slightly higher at the cleavage stage than at the blastula stage, and reached the maximum level during the zoea stage of embryos. The minimum level of *MnGus* expression occurred during the perinucleolus stage in the ovary, while the maximum was at the oil globule stage, and then the level of *MnGus* expression gradually decreased with the advancement of ovarian development. The expression level of *MnGus* in muscle was much higher than that in other tissues in mature prawn. The gustavus cDNA sequence was firstly cloned from the oriental river prawn and the pattern of gene expression was described during oocyte maturation, embryonic development, and in other tissues. The differential expression patterns of *MnGus* in the embryo, ovary and other somatic tissues suggest that the gustavus gene performs multiple physiological functions in the oriental river prawn.

## Introduction

It is well known that embryonic development originates from the formation of gametes that are responsible for transmitting genetic information from one generation to the next. In many organisms, the primordial germ cells (PGCs) are specialized and set apart from the somatic cells during early development. However, in other cases, PGCs represent the earliest cell lineage to be determined and finally they arrive at the gonad and differentiate into gametes [1]. The formation of germ cell precursors depends on a specialized cytoplasm known as germ plasm, which contains RNAs and proteins that are required for embryonic patterning and germ cell formation [2], [3], [4], [5]. The specification, differentiation, and migration of PGCs are governed by a tightly controlled series of gene expression events [6]. The VASA gene has been identified in the fly *Drosophila* and it encodes a DEAD (Asp-Glu-Ala-Asp) box family protein [7]. This gene is a putative RNA helicase and is present both in polar granules at the posterior end of the oocyte and in the nuage structure of germ cells [8], [9].

Recently, a protein termed gustavus (GUS) which interacts with VASA has been identified in the fly *Drosophila* and this protein belongs to SPRY domain- and SOCS box-containing (Spsb) family which contains two conserved protein domains: the SPRY (sp1A/ryanodine receptor) domain and SOCS (suppressor of cytokine signaling) box [10], [11]. This gene is required for localizing pole plasm and specifying germ cells [10], [12]. Mutations in the GUS gene may result in a sterile female phenotype in *Drosophila* [10]. The SPRY domain exists in ryanodine receptors and is thought to mediate Ca^2+^ release from the sarcoplasmic reticulum [13]. The SOCS box is a sequence motif identified in the suppressor of cytokine signaling [14], [15], which is associated with ubiquitination of proteins for proteasomal degradation [16].

The oriental river prawn *Macrobrachium nipponense* is an important species for freshwater aquaculture in China. However, in captivity, this species has a tendency of early maturation, thus negatively impacting on growth [17]. To understand the controlling mechanism of sex maturation, a high-quality cDNA library for this species has been established from ovary tissue [17]. In a recent study, Zhang et al. [18] found that an ubiquitin-conjugating enzyme (Ubc9) contributed to the controlling mechanism of the embryogenesis and oogenesis of *M. nipponense* although the sex maturation of this species is through the regulation of multiple genes. To further understand the molecular mechanism of maturation, other gene regulations involved in the developmental process also need to be examined in *M. nipponense*. In a recent study, the gustavus gene has been expressed in the ovaries of *Drosophila melanogaster* at all stages suggesting its role in the regulation of oogenesis [12]. Thus, it is hypothesized that the homologue of the gustavus gene controls the oogenesis and development in crustacean. In this study, we cloned and characterized the gustavus homologue from a crustacean and examined the expression pattern of the cloned gene in the developing embryo, ovary and other tissues.

## Materials and Methods

### Ovary, Embryo and Tissue Collections

Female oriental river prawns at different developmental stages were obtained from a commercial farm near Shanghai, China. The prawns were maintained in aerated freshwater for 72 h prior to tissue collections between March and July, 2008. After prawn spawning, the development of ova was continuously monitored. Each developmental stage of ovaries and embryos was determined following the criteria by Gao et al. [19], Zhao et al. [20] and Zhang et al. [18]. Muscle, blood, heart, hepatopancreas, testis, thoracic ganglion, and gill tissues were also collected from mature prawns. The samples were treated with phosphate buffer saline (PBS), and then preserved in liquid nitrogen at −80°C before RNA extraction.

### RNA Isolation and Reverse Transcription (RT)

Total RNA was extracted from ovaries and embryos at different stages of development and was also from other tissues in mature prawns with Unizol Reagent (Biostar, Shanghai, China) in accordance with the protocol of the manufacturer. The isolated RNA was treated with RNase-free DNase I (Sigma) to eliminate possible genomic DNA contamination. The concentration of each total RNA sample was then measured by spectrophotometry (Eppendorf), and quantified prior to the reverse transcription reaction.

The first-strand cDNA synthesis in RT was performed using a PrimScript^TM^ first strand cDNA synthesis kit (TaKaRa) to transcribe poly (A)^+^mRNA with oligo-dT primers under the manufacturer's recommended conditions. The cDNA was kept at −20°C for real-time quantitative PCR (RT-QPCR).

### 5′- and 3′-RACE Amplification of *MnGus* Gene

Based on the homology to *Drosophila* and other organisms, we identified the GUS-related genes in the cDNA library constructed with the previtellogenesis ovary tissues. The total RNA from previtellogenesis ovary tissue was used as a template to amplify the cDNA ends of *MnGus*. All primers used in this study are shown in Table 1. The two primers of GUS-F and GUS-R validated the *MnGus* fragments from the EST cDNA library. The 3′-RACE (3′-RACE System, Invitrogen, Carlsbad, CA, USA) and 5′-RACE (Smart Race Kit, Clontech, Palo Alto, CA, USA) methods were subsequently carried out according to the manufacturer's instructions. The gene-specific primers of *MnGus* were designed based on the known fragments. First of all, AP (as the RT primer), AUAP (as the universal amplification primer), the two gene-specific primers of GUS-3GSP1 (as the 3′RACE first primer) and GUS -3GSP2 (as the nested primer) were used for 3′-RACE. The 5′-RACE including RT, dC tailing and PCR amplifications was carried out following the protocol provided by the manufacturer. The primers, UPM (as the first amplification primer), NUP (as the nested primer) and primer GUS-5GSP1 or GUS-5GSP2 were used respectively for 5′-RACE. The touchdown-PCR reactions were carried out at 94°C pre-denaturation for 3 min, then 94°C for 30 s, 68°C for 30 s, and 72°C for 1 min in the first cycle and the anneal temperature decreased 1°C per cycle. After 11 cycles, conditions were changed to 94°C for 30 s, 57°C for 30 s, and 72°C for 1 min for 19 cycles. The duration of 72°C elongation was 7 min.

**Table 1:** Universal and specific primers used in this study.

Name	Sequence (5′-3′)
GUS-F	CTGGTCTACTCGGCAACG
GUS-R	ATCTGGGACAACGAAGGT
GUS-3GSP1	AATACACTGGTCTACTCGGCAACGG
GUS-3GSP2	CAGGGTTTGGTAGGCAGTAATGAC
GUS-5GSP1	GCTTGCCGTATTATCCAGCGACA
GUS-5GSP2	CCCGTTGCCGAGTAGACCAGTGTA
GUS-RTF	TGATGGATTCATGTCGCTGG
GUS-RTR	GTTCGGATGTCACGTTGGTG
β-actin-RTF	AATGTGTGACGACGAAGTAG
β-actin-RTR	GCCTCATCACCGACATAA
AP	GGCCACGCGTCGACTAGTACTTTTTTTTTTTTTTTTT
AUAP	GGCCACGCGTCGACTAGTAC
UPM	Long:CTAATACGACTCACTATAGGGCAAGCAGTGGTATCAACGCAGAGTShort:CTAATACGACTCACTATAGGGC
NUP	AAGCAGTGGTATCAACGCAGAGT

### Subcloning and Sequencing

The PCR fragments were subjected to electrophoresis on a 1.5% agarose gel to compare the length difference. Amplified cDNA fragments were cloned into the pMD18-T vector (TaKaRa) following the manufacturer's instructions. Recombinant bacteria were identified by blue/white screening and confirmed by PCR. At least two positive colonies were sequenced by the dideoxy chain termination using an automatic DNA sequencer (ABI Applied Biosystems Model 3730).

### Nucleotide Sequence and Bioinformatics Analyses

Sequences were analyzed based on nucleotide and protein databases using the BLASTX and BLASTN program (http://www.ncbi.nlm.nih.gov/BLAST/). The protein prediction was performed using the ORF Finder tool (http://www.ncbi.nlm.nih.gov/gorf/gorf.html). The SOCS and SPRY domains were defined by NCBI conserved domain search (http://www.ncbi.nlm.nih.gov/structure). Multiple sequence alignments of GUS proteins were carried out online (http://www.ch.embnet.org/software/BOX_form.html). Phylogenetic relationships were deduced and dendrograms were produced using the MEGA 4.0 program (http://www.megasoftware.net/).

### Real-time Quantitative PCR Analysis of *MnGus*

RT-QPCR was carried out by the Stepone Plus real-time PCR detection system (Applied Biosystems). The first strand cDNAs from different samples were used separately as a template in a 20 µL PCR reaction in a power SYBR green PCR master mix (PE Applied Biosystems). Three samples were collected from each developmental stage and each sample was measured in duplicate. The reaction mixture was initially incubated for 15 min at 95°C to activate the HotStart Taq DNA polymerase, then amplified by 40 cycles of denaturation at 95°C for 15 s. Annealing and extension were at 60°C for 60 s. Amplification of β-actin as an internal reference was also carried out in the same sample (the primer's sequences are shown in Table 1). DEPC-water for the replacement of template was used as a negative control. A relative standard curve was developed using 10-fold serial diluted cDNA. The standard curves were included in all runs in order to relate to quantitative data. The concentration of cDNA in each sample was calculated from the standard curve. A melting curve analysis of amplification products was performed at the end of each PCR reaction to confirm that only one PCR product was amplified and detected.

### Statistical Analysis

As for the results of RT-QPCR, observations at the each developmental stage were calculated to derive the mean and standard error (SE). All data obtained from the RT-QPCR analysis were log transformed before using data analysis with one-way ANOVA. When the treatment difference was significant, a post hoc test was used for multiple comparisons (Duncan test). Differences were considered significant at *P*<0.05 and highly significant at *P*<0.01.

## Results

### *MnGus* cDNA Sequence of *M. nipponense*

**Figure 1:**
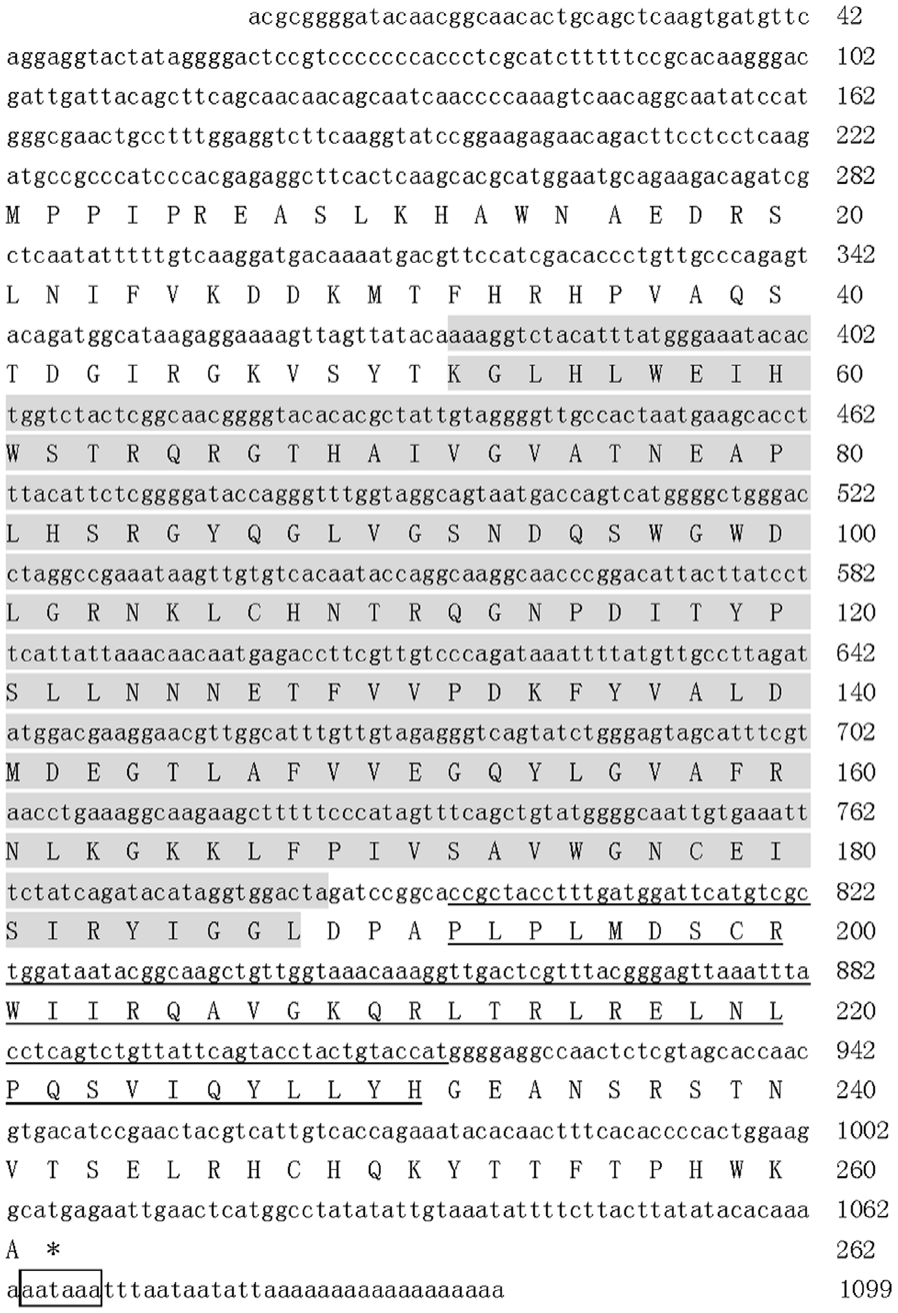
cDNA sequences and deduced amino acids of *MnGus* from *M. nipponense*. The nucleotide sequence is displayed in the 5′-3′ directions and numbered to the right. The deduced amino acid sequence is shown in a single letter amino acid code. Codons are numbered at the right with the methionine initiation codon and the termination codon denoted by asterisk. The termination signal in the 3′-untranslated region (UTR) is highlighted by frame. The SPRY domain is shadowed in the N-terminal and SOCS box is underlined in the carboxy-terminal. This is determined based on the motif scan at http://hits.isb-sib.ch/cgi-bin/motif_scan and Blast p at http://blast.ncbi.nlm.nih.gov/Blast.cgi.

In this study, the full-length cDNA of *MnGus* was obtained and deposited in GenBank (Accession No. GU462157). The *MnGus* cDNA was 1099 bp in length with 5′ and 3′ non-translated regions, poly-A tail, and a 786 bp ORF encoding a 262-amino-acid protein (Fig. 1).

### Bioinformatics Analysis

The analysis of *MnGus* protein revealed that it comprised 262 amino acids with a predicted molecular weight of 29.70 kDa and an isoelectric point of 9.32. It contained conserved-protein domains of a SPRY domain and a SOCS box through scanning motif at http://hits.isb-sib.ch/cgi-bin/motif_scan and Blast p at http://blast.ncbi.nlm.nih.gov/Blast.cgi.

**Figure 2:**
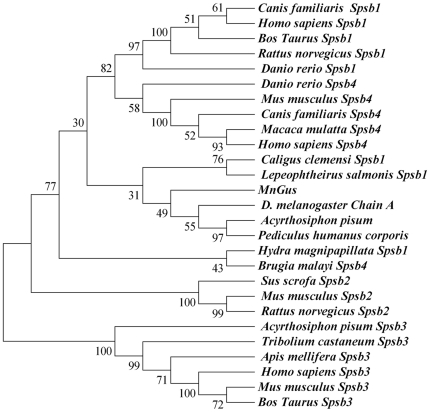
The phylogenetic tree of gustavus from different organisms based on the comparisons of amino acid sequences. Species names and types of Spsb are listed on the right of the tree. Their accession number in GenBank as followed: *Canis familiaris* Spsb-1 (XP 536736); *Homo sapiens* Spsb-1 (Q96BD6); *Bos Taurus* Spsb-1 (NP001029381); *Rattus norvegicus* Spsb-1 (NP 001101464); *Danio rerio* Spsb-1 (NP 001020631); *Danio rerio* Spsb-4a (XP 686153); *Mus musculus* Spsb-4 (NP 660116); *Canis familiaris* Spsb-4 (XP 542811); *Macaca mulatta* Spsb-4 (XP 001113018); *Homo sapiens* Spsb-4 (Q96A44); *Caligus clemensi* Spsb-1 (ACO15582); *Lepeophtheirus salmonis* Spsb-1 (ACO13126); *Drosophila melanogaster* Chain A (2IHS-A); *Pediculus humanus corporis* (EEB17985); *Brugia malayi* Spsb-4 (XP 001900109); *Hydra magnipapillata* Spsb-1 (XP 002160204); *Sus scrofa* Spsb-2 (XP 001928484); *Mus musculus* Spsb-2 (O88838); *Rattus norvegicus* Spsb-2 (Q5M877); *Acyrthosiphon pisum* Spsb-3 (XP 001949481); *Tribolium castaneum* Spsb-3 (XP 966919); *Apis mellifera* Spsb-3 (XP 001122547); *Homo sapiens* Spsb-3 (Q6PJ21); *Mus musculus* Spsb-3 (Q571F5); *Bos Taurus* Spsb-3 (Q3MHZ2).

Multiple protein alignments showed that *MnGus* protein had homology to the known and predicted Spsb homologues in other animals. For example, the *MnGus* protein shared 73% identity with the GUS of *Acyrthosiphon pisum* (Accession No. XP001951784), 62% identity with the Spsb1 of *Caligus clemensi* (Accession No. ACO15582), and 53% identity with the Spsb1 of *Lepeophtheirus salmonis* (Accession No. ACO13126). A phylogenetic tree was constructed based on the neighbor-joining method using the complete Spsb proteins deposited in NCBI by MEGA 4.0. The tree showed that *MnGus* was lined with the Spsb1 group of Arthropoda such as *C. clemensi* and *L. salmonis*, but separated from its homologues of Vertebrata Spsb1 and Spsb4. Its relationship to Spsb2 and Spsb3 was more distant than Spsb1 and Spsb 4 (Fig. 2). The results of bioinformatics analysis suggested that the *MnGus* should belong to a Spsb1 homologue.

### Gene Expression of *MnGus*

**Figure 3:**
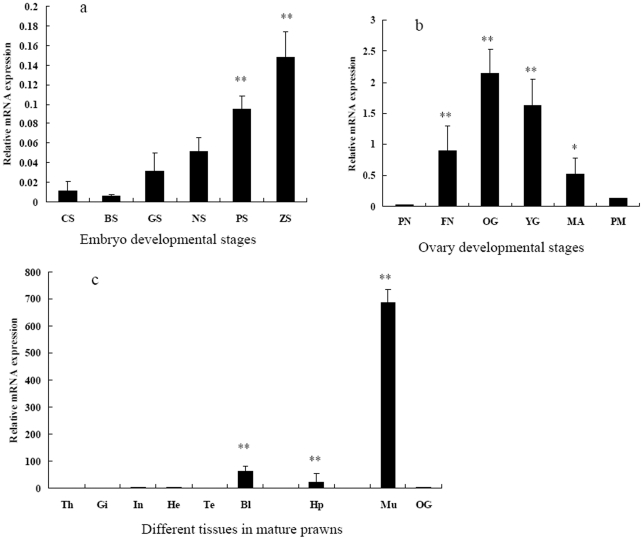
The expression profiles of *MnGus* at different development stages of the embryo and the ovary, and in other tissues revealed by RT-QPCR. The amount of *MnGus* mRNA was normalized to the β-actin transcript level. Data are shown as means ± SE (standard error) of three replicates in the ovary, embryo and other tissues. The “y” axis represents the relative expression of the *MnGus*/β-actin mRNA ratio. The asterisks indicate significant difference by comparing with the CS, PN and OG stages in the embryo, ovary and other tissues respectively. The single asterisk (*) indicates a significant difference at *P*<0.05, and the double asterisk (**) indicates the level of difference was at *P*<0.01. CS- cleavage stage; BS- blastula stage; GS- gastrul stage; NS- nauplius stage; PS- protozoea stage; ZS- zoea stage; PN- perinucleolus; FN- fusion nucleolus; OG- oil globule; YG- yolk granule; MA- maturation; PM- paracmasis; Th- thoracic ganglia; Gi- gill; In- intestines; He- heart; Te- testis; Bl- blood; Hp- hepatopancreas; and Mu- muscle.

RT-QPCR results showed that the *MnGus* expression level changed significantly over the course of embryonic and ovary developments. In the embryonic stage, the expression of *MnGus* was slightly stronger in the cleavage stage (CS) than in the blastula stage (BS). After decreasing to a low level, the expression of *MnGus* gradually increased with the development of the embryo and peaked at the zoea stage (ZS) (Fig. 3a). In the ovary, the expression of *MnGus* was lowest at the perinucleolus (PN) stage, but increased abruptly at the fusion nucleolus (FN) stage and reached the maximum at the oil globule (OG) stage. Subsequently, it then gradually decreased from the yolk granule (YG) stage to the paracmasis (PM) stage (Fig. 3b). To scrutinize the biological functions of *MnGus*, the expression patterns in the thoracic ganglia, gill, intestine, heart, testis, blood, hepatopancreas and muscle tissues were also examined. Interestingly, the *MnGus* expression in the muscle was much higher than in other tissues (Fig. 3c).

## Discussion

In this study, for the first time we identified that the homologue gustavus (GUS) gene of the oriental river prawn belongs to the Spsb gene family and its sequence was found to be similar to the gustavus gene of *Drosophila* and other organisms. The predicted protein carried a SPRY domain in the N-terminal and a SOCS box in the carboxy-terminal. Currently, four kinds of Spsb families (Spsb-1, -2, -3, -4) have been identified in mice [12], [21], humans [22], [23], and zebra fish [6]. Spsb1 and Spsb4 can bind strongly to both prostate apoptosis response protein-4 (Par-4) and VASA peptides, whereas structural changes limit high affinity interactions with Spsb2 to these aspartate-containing sequences [24]. Through interacting with the N-terminal region of inducible nitric oxide synthase (iNOS), Spsb2 can recruit an E3 ubiquitin ligase complex to polyubiquitinate iNOS, resulting in its proteasomal degradation [25]. Our analysis of the protein showed that the *MnGus* was similar to Spsb1 of other animals. We therefore deduce that the *MnGus* belongs to the Spsb1 family. Moreover, the fact that Spsb1 is highly conserved in vertebrates and invertebrates suggests that the GUS is functionally important in a variety of organisms.

GUS, like VASA and other proteins involved in oocyte patterning, is a component of cytoplasmic ribonucleic proteins (RNPs) [10]. The RNP complexes have a mechanism to generate cell polarity and can provide the basis for patterning during embryonic development [26]. Early embryonic development of an organism is controlled by maternal factors [27]. In our study, the expression of *MnGus* was detected in the early stage of *M. nipponense* embryos, but decreased at the blastula stage (BS), suggesting that *MnGus* mRNA is possibly derived from a maternal source during oogenesis. A similar expression pattern of Spsb was found in the developmental embryos of zebrafish [6], but as the embryo developed, the *MnGus* transformed to self-expression during the blastula stage (BS) and zoea stage (ZS). The expression level of *MnGus* obviously increased at the protozoea stage (PS) and peaked at the zoea stage (ZS) when organogenesis, especially myogenesis, was most active. This implies that *MnGus* may play an important role in the organogenesis of *M. nipponense* in late embryonic development and this also supports the result of high *MnGus* expression (i.e., Spsb1 family protein) in muscles. However, this result was at odds with a previous study in mouse which reported that Spsb3 was highly expressed in muscles, rather than Spsb1 [28]. The function of the Spsb family is entirely unknown [28], therefore further research should be directed to scrutinize its biological function in the muscular activity of the oriental river prawn since Spsb proteins may regulate multiple target proteins [29].

The developmental process of oocytes in prawn undergoes a series of cellular differentiations in which differential genes are expressed temporally and spatially to ensure the proper development of oocytes [30], [31]. VASA is a key protein for the translational activity of the *Drosophila* pole plasm accumulated at the posterior pole of a developing oocyte, and GUS is essential for the posterior localization of VASA [10], [32]. The latest research [33] has shown that Cullin-5 phenotypes are strongly enhanced by reduced activity of GUS which encodes a substrate receptor of Cul-5-based ubiquitin E3 ligases in *Drosophila* oogenesis. The Cul-5/*Gus* ubiquitin E3 ligases participate in the ovarian tissue morphogenesis, germ cell proliferation and maintenance of ovarian germ cells. In our study, the expression level of *MnGus* varied significantly during ovary development. The lowest expression of *MnGus* was at the perinucleolus (PN) stage when the number of previtellogenic oocytes increased. In contrast, the highest expression occurred at the oil globule (OG) stage when the size of vitellogenic oocytes substantially increased. These results suggest that *MnGus* is possibly involved in the complex event of maturation of oocytes. Nevertheless, the interactions between *MnGus* and the target proteins (e.g., VASA, Cullin-5 or other proteins) require further study.

In conclusion, *MnGus* plays a critical and complex role in the embryogenesis and oogenesis of *M. nipponense*. This study advances our understanding of the multiple biological functions of the gustavus gene. Moreover further work should be focused on the understanding of the gustavus functions in crustacean.
